# Controllable One-Step Synthesis of Mixed-Phase TiO_2_ Nanocrystals with Equivalent Anatase/Rutile Ratio for Enhanced Photocatalytic Performance

**DOI:** 10.3390/nano11051347

**Published:** 2021-05-20

**Authors:** Yuchen Lei, Yun Yang, Peilin Zhang, Jiaojiao Zhou, Jing Wu, Kuang Li, Weiwei Wang, Luyang Chen

**Affiliations:** Key Laboratory for Ultrafine Materials of Ministry of Education, School of Materials Science and Engineering, East China University of Science and Technology, Shanghai 200237, China; y30180267@mail.ecust.edu.cn (Y.L.); 18616819506@163.com (Y.Y.); ZPL18217735506@163.com (P.Z.); Y10190102@mail.ecust.edu.cn (J.Z.); Y30180472@mail.ecust.edu.cn (J.W.); likuang23@163.com (K.L.); 18717773018@163.com (W.W.)

**Keywords:** TiO_2_ nanocrystals, photocatalytic degradation, mixed crystal phase, hybrid structure

## Abstract

In this study, the novel mixed-phase TiO_2_ nanocrystals (s-TiO_2_) with nearly equivalent anatase/rutile ratio were fabricated in the reagent of sec-butanol at the relatively low temperature of 80 °C by using a facile one-step condensing reflux method. The photocatalytic water splitting hydrogen production performance of s-TiO_2_ nanocrystals is close to that of commercial TiO_2_ (P25), and its photocatalytic degradation performance is about four times that of P25. The energy-level staggered interfaces and surface bridged hydroxyl groups significantly increased due to the anatase/rutile mixed-phase crystal structure and high specific surface area, which might generate the synergistic effect for the improvement of photocatalytic degradation.

## 1. Introduction

As only a small number of harmful pollutants can be degraded by the self-cleaning ability of the environment, the treatment of pollutants has always been one of the important research issues [1,2,3,4]. Many methods have been explored for controlling pollutants, such as adsorption, biochemistry, sedimentation, and so on. Among them, photocatalytic degradation can convert pollutants into completely harmless or less toxic organic compounds under mild reaction conditions. The basis of photocatalytic technology lies in the unique electronic structure of semiconductor materials. The photocatalytic degradation is essentially a typical redox process. According to the solid energy band theory, a semiconductor catalyst undergoes an electronic transition under light conditions to generate photogenerated electron–hole pairs. Then, the electron–hole pairs directly oxidize and reduce pollutants, or further generate strong oxidizing hydroxyl radicals on the surface to degrade pollutants [5,6,7,8]. The photocatalyst with broad spectrum and long life can be the ideal candidate for the pollutant degradation [4,9].

Titanium dioxide (TiO_2_) has high valence band and conduction band potential, which implies that its photogenerated carrier redox performance is strong [10]. Moreover, it has a high chemical stability, corrosion resistance, non-toxicity, and low cost. Therefore, it has been widely studied as a traditional photocatalyst. However, the wide bandgap causes its light absorption range to be in the ultraviolet region, which accounts for only about 4% of solar energy [11]. The high recombination probability of photogenerated electrons and holes results in the low quantum efficiency [12,13,14]. Recently, to improve the catalytic activity in the sunlight, TiO_2_ is mainly modified via a variety of methods, including ion doping, semiconductor coupling, precious metal supporting, and dye sensitization [15,16,17,18,19]. Among them, semiconductor coupling can construct interfaces with interleaved energy levels by using their different energy band structure. Due to the energy level difference between semiconductors, photogenerated carriers can transfer between semiconductors, which facilitates the separation of carriers for redox reaction.

TiO_2_ has three crystal phases (rutile/anatase/brookite) with different bandgaps. Studies have shown that the photocatalytic activity of mixed crystal phase TiO_2_ can be significantly improved in comparison with single-phase TiO_2_ [20,21,22,23]. The synergy effect between the different phases is the reason for the enhanced photocatalytic activity of mixed-phase TiO_2_. The interlaced bandgaps between the interfaces contribute to charge separation and the photogenerated electrons tend to flow from the rutile phase to the anatase phase [14]. For example, Xu and co-workers developed anatase and rutile dual-phase TiO_2_ nanofibers with stronger photocatalytic hydrogen production performance [24]. Furthermore, fully mixed anatase/rutile nanocrystals with mass ratio of 1:1 seem to form more energy-level staggered interfaces, which can greatly promote the separation of photogenerated carriers. However, in the most mixed-crystal studies, the component of rutile phase is far lower than that of the anatase phase [25,26,27,28,29,30,31]. Meanwhile, it is usually necessary to prepare the rutile-rich mixed nanocrystals under the high temperature due to the more stable rutile phase irreversibly transformed from the anatase phase, which means that industrial production will be faced with the disadvantages of high energy consumption. Therefore, it is urgent to explore a low-temperature route to synthesize TiO_2_ mixed-phase nanocrystals [32].

Additionally, many studies have confirmed that the higher catalytic activity strongly depends on the smaller particle size due to the larger specific surface area [14]. The photocatalytic activity of nanostructured CdS, ZnS, PbS, TiO_2_-Al_2_O_3_, and other semiconductor particles is also significantly better than the counterpart bulk materials [33]. It is generally believed that the improved specific surface area leads to an increase in the number of active sites [14]. In addition, the distance for the charge to move from the generation site to the surface will also be shortened to participate in the reaction, which implies that the charge separation effect can be better [14]. When the particle size is less than 10 nm, the size effect of the TiO_2_ nanocrystals results in the increase of bandgap, which in turn makes the conduction band potential more negative or the valence band potential more positive, thereby enhancing the redox ability of photogenerated carriers [14,20].

Herein, the synthesis of novel TiO_2_ nanocrystals with rutile-rich mixed-phase structure and small particle size via a simple one-step condensing reflux method at a relatively low temperature of 80 °C are reported. The crystal phase ratio of anatase/rutile close to 1:1 is beneficial to the separation of carriers, and the large specific surface area with many active sites can contribute to the occurrence of catalytic reactions. The product exhibits nearly four times improved photocatalytic degradation performance in comparison with commercial TiO_2_ (designated as P25).

## 2. Materials and Methods

### 2.1. Materials

Titanium tetrachloride (TiCl_4_) and chloroplatinic acid hexahydrate (H_2_PtCl_6_·6H_2_O) were purchased from Macklin (Shanghai, China), oleic acid, sec-butanol, tert-butanol, triethanolamine, ethanol, and sodium sulfate (Na_2_SO_4_) were supplied by General-Reagent (Shanghai, China), titanium dioxide P25 was sourced from Innochem (Beijing, China), nafion 117 was purchased from Sigma-Aldrich (Shanghai, China), and rhodamine B was supplied by D&B (Shanghai, China).

### 2.2. Synthesis of TiO_2_ Nanocrystals via Two Kinds of Butanol Reagents

Twenty-three mL of sec-butanol or tert-butanol was measured as two different solvents. Under ice bath conditions, 1 mL TiCl_4_ was transferred to different solvents with a pipette gun in a fume cupboard. After the mixed solution was magnetically stirred for 5 min, 5 mL of oleic acid was added. The above solution was continuously stirred for 5 min and then transferred to a round bottom flask, then condensed and refluxed for 3 h in an oil bath at 80 °C. Subsequently, the product was rinsed several times with ethanol and deionized water, respectively. Finally, the as-prepared sample was dried in a vacuum oven at 60 °C. For the convenience of expression and comparison, the TiO_2_ nanocrystals obtained from the sec-butanol and tert-butanol alcohol system are designated as s-TiO_2_ and t-TiO_2_, respectively.

### 2.3. Microstructure Characterizations

The morphology crystal lattice of the sample was clearly observed by a transmission electron microscope (TEM) (JEM-2100, Tokyo, Japan). D/max2550VB/PC (Tokyo, Japan) was used for X-ray powder diffraction (XRD) pattern characterization. The Brunauer–Emmett–Teller (BET) technique (Artochem II 2920, Norcross, GA, USA) was used to determine the specific surface area and the adsorption–desorption curve. X-ray photoelectron spectroscopy (XPS) analysis was performed using a Thermo Fisher Scientific Escalab 250Xi spectrometer (Abingdon, UK). With 350 nm as the excitation wavelength, the photoluminescence spectrum (PL) was measured with PE-LS-55 (Shenzhen, China) at room temperature. UV-9000s (Shanghai, China) were used to measure the ultraviolet-visible diffuse reflection spectrum (DRS). The Raman test was performed with a laser micro-Raman spectrometer (Gloucestershire, UK) (≤10.2/cm/invia reflex).

### 2.4. Photocatalytic Degradation Experiments

Thirty mg of photocatalyst was added to a glass petri dish containing 30 mL of deionized water. The solution was sonicated for one hour to achieve uniform dispersion, and then dried at 80 °C for three hours to evaporate water. Thirty mL rhodamine B (RhB) solution (4.8 mg/L) was added. Then, the petri dish was placed in the dark for one hour to achieve solid–liquid adsorption equilibrium, and then irradiated under a 300 W xenon lamp (λ = 365^±10^ nm). Every 20 min, 2.2 mL of the solution in the petri dish was taken out, centrifuged to remove the solid catalyst, and then measured with an ultraviolet-visible spectrophotometer. Since the concentration was proportional to the absorbance, the photocatalytic degradation curve of the catalyst was obtained.

### 2.5. Photocatalytic Decomposition of Water to Produce Hydrogen Test

To the quartz reactor, 30 mg of photocatalyst, 10 mL of triethanolamine, and 90 mL of deionized water were added, and then 240 μL H_2_PtCl_6_·6H_2_O solution (10 g/L) was added by a pipette. After stirring, the reactor was connected to the semi-manual injection photocatalytic hydrogen production system. A 300 W xenon lamp (λ = 365^±10^ nm) was used to irradiate the reactor for 1 h to achieve 3 wt% precious metal deposition. Then, the reactor was continuously irradiated under a 300 W xenon lamp (λ > 420 nm). The gas of Ar was used for degassing. During this period, a 6 °C cooling water circulation device was placed outside the reactor to condense water vapor and offset the temperature increase caused by long-term strong-light irradiation. A fixed amount of gas was injected every 30 min, and the amount of H_2_ generated on the online gas chromatograph GC 2060 was measured. After 4 h of continuous irradiation, a total of eight cumulative hydrogen production amounts were obtained to draw images of continuous photocatalytic hydrogen production.

### 2.6. Photoelectrochemical Measurements

The photoelectrochemical measurements are executed by a three-electrode system. That is, a 1.0 × 1.0 cm Nafion FTO glass with catalyst coating was used as the working electrode, Pt was used as the counter electrode, and a saturated Ag/AgCl electrode was used as the reference electrode. Then, the photoelectric response test and impedance test were performed with a CHI760E under a 300 W Xenon lamp (λ > 420 nm).

## 3. Results and Discussion

### 3.1. Basic Structure Characterization of Photocatalyst

**Figure 1:**
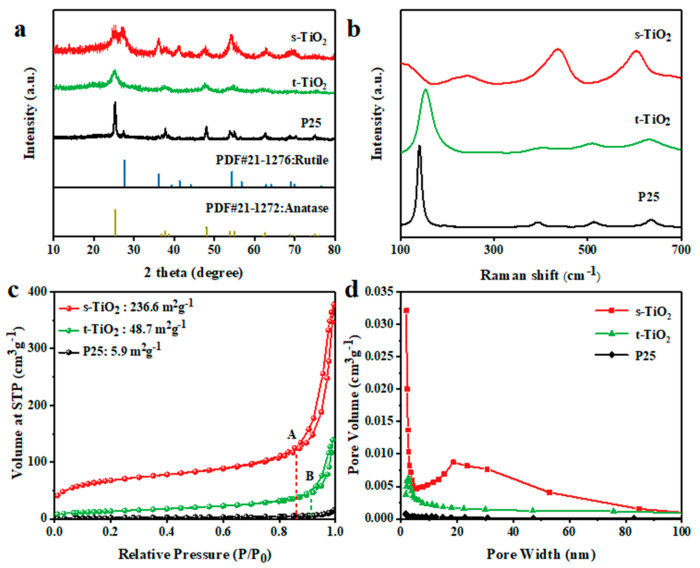
(**a**) XRD patterns, (**b**) Raman spectra, (**c**) BET spectrum, and (**d**) pore size distribution of s-TiO_2_, t-TiO_2_, and P25.

The phase analysis and particle size evaluation can be investigated by XRD. As shown in Figure 1a, it can be seen that s-TiO_2_ has two crystalline phases, including rutile and anatase phase, while t-TiO_2_ is only composed of anatase phase. Compared to the commercial TiO_2_ (P25), the diffraction peak of s-TiO_2_ is obviously broadened due to the very small particle size. According to Equation (1), the mass ratio of anatase phase to rutile phase of s-TiO_2_ can be calculated to ~48/52, which is evidently different from ~79/21 of P25 [24].

F_R_ = I_R_/(0.886I_A_ + I_R_) (1)

F_R_ represents the mass fraction of the rutile phase, while I_A_ and I_R_ stand for the integrated intensity of diffraction peak of the anatase (101) plane and the rutile (110) plane, respectively.

Then, the average crystallite size of nanocrystals can be estimated from the Scherrer Equation [17]:

D = Kγ/Bcosθ(2)

In Equation (2), D represents crystallite size, K = 0.9 is the Scherrer constant, and γ is the X-ray wavelength, when the anode target is a copper target, γ = 0.154056 nm. B represents the half-width of the diffraction peak and θ is the diffraction angle. The calculation results are shown in Table 1. The diameters of s-TiO_2_ nanocrystals with rich rutile are far smaller than those of P25 particles.

**Table 1:** Phase component and specific surface areas of s-TiO_2_, t-TiO_2_, and P25.

TiO_2_	Phase Composition (wt%)		Crystalline Sizes (nm)	
	Anatase	Rutile	Anatase	Rutile
s-TiO_2_	48	52	7.82	6.39
t-TiO_2_	100	/	4.46	/
P25	79	21	14.4	13.9

**Table 2:** Microstructural characteristic and rate constants of mixed-phase TiO_2_.

Raw Materials and Preparation Method	Phase Composition (wt%)		S_BET_ (m^2^g^−1^)	Reference
	Anatase	Rutile		
s-TiO_2_	48	52	236.6	This study
P25	79	21	5.9	/
TiOSO_4_ + H_2_O + H_2_SO_4_	77.4	22.6	32.2	[25]
TTIP + ethanol + xylene flame spray pyrolysis	88	11	249	[26]
TTIP + acetonitrile + xylene flame spray pyrolysis	92	8	36	[29]
TTIP + isopropanol + HF + TiCl_3_	79	21	/	[30]
TiCl_4_ + air + C_2_H_4_ laser pyrolysis	46	54	/	[32]
TiCl_3_ + HNO_3_ + ethanol + urea	59	41	/	[34]
TiO_2_ + H_2_O oven	44	56	/	[35]

The Raman spectrum can also analyze the mixed crystal phase structure in TiO_2_ nanocrystals. As shown in Figure 1b, t-TiO_2_ has four distinct bands at 149, 400, 520 (a doublet), and 637 cm^−1^, assigned to the fundamental E_g_, B_1g_, A_1g_, and B_2g_ and E_2g_ modes of anatase-phase TiO_2_, respectively. The two typical modes of rutile-phase TiO_2_ (B_1g_, B_2g_) are located at 448 and 612 cm^−1^, which are obviously reflected in s-TiO_2_ [36]. The lack of obvious rutile phase peak in P25 may be attributed to the low content of rutile. The Raman modes of TiO_2_ phase become difficult to be discriminated when the nanocrystal size drops below 20 nm due to the mode displacement and convolution [32]. In addition, compared with P25, the increase in the proportion of rutile in the s-TiO_2_ mixed crystal caused an increase in the red shift of its anatase phase at 149 cm^−1^. This change may stem from internal defects related to oxygen vacancies and Ti^3+^ in the crystal [30], or the influence of the rutile phase in the mixed structure. The rutile phase composition of s-TiO_2_ is higher than that of P25, and the red shift of E_g_ should be more obvious.

The BET analysis can be utilized to analyze the specific surface area and pore size distribution of materials. As shown in Figure 1c, the BET surface area of s-TiO_2_ with ~236.6 m^2^ g^−1^ is far larger than those of t-TiO_2_ (48.7 m^2^ g^−1^), P25 (5.9 m^2^ g^−1^), and other reported TiO_2_ nanocrystals (Table 1 and Table 2), which should be ascribed to the nano-scaled size of the TiO_2_ nanocrystals. Moreover, the BET curves of s-TiO_2_ and t-TiO_2_ both show V-shaped curves with H3 hysteresis ring. The samples have weak adsorption with N_2_ in the low-pressure range from 0.05 to 0.35. When the relative pressure is close to zero, the N_2_ adsorption volume of s-TiO_2_ still can reach 41 cm^3^ g^−1^, indicating that there exist many micropores less than 2 nm in s-TiO_2_. After the relative pressure is greater than 0.85, the adsorption volumes of s-TiO_2_ and t-TiO_2_ rise sharply because the samples contain a considerable amount of mesopores (2~50 nm) and macropores (>50 nm). H3 hysteresis loop is given by flaky particulate materials such as clay, or by fractured pore materials [37]. Judging from the type of hysteresis loop, the isotherm has no obvious saturated adsorption platform, which means that the pore structure is irregular, including flat slit holes, cracks, and wedge structures [38]. According to the pore size distribution curve (Figure 1d), the s-TiO_2_ sample contains a large number of micropores with a diameter of less than 2 nm. We infer that the hysteresis loop can mainly be attributed to these very small irregular pores, which probably exist between the grain boundary of nanocrystals. Since s-TiO_2_ contains two crystal phases, the possibility of forming grain boundary defects is greatly improved. Therefore, the pore size distribution discloses that the number of micropores in s-TiO_2_ is significantly increased in comparison with single-phase t-TiO_2_. In addition, the onset of the s-TiO_2_ hysteresis ring (point A) is lower than that of the t-TiO_2_ hysteresis ring (point B), which indicates that the mesopore structure in s-TiO_2_ has a wider pore size distribution. As shown in Figure 1d, the bimodal pore distribution can be observed in s-TiO_2_. The pore volume of micropores, mesopores, and macropores in s-TiO_2_ far exceeds that of t-TiO_2_ and P25, but the mesopores occupy the main proportion of pore structure for s-TiO_2_. The great dispersibility of s-TiO_2_ can contribute to its largest specific surface area among three samples.

**Figure 2:**
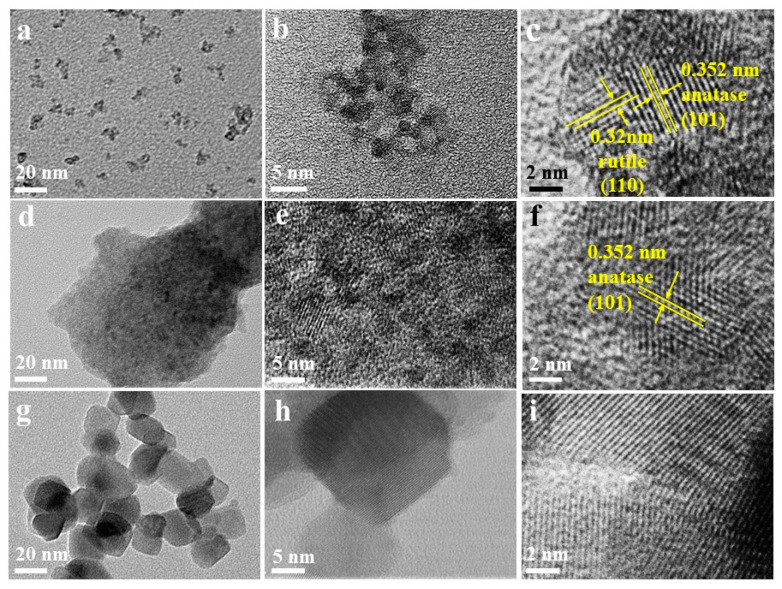
TEM images of (**a**–**c**) s-TiO_2_, (**d**–**f**) t-TiO_2_, and (**g**–**i**) P25.

The microstructure of TiO_2_ was investigated by TEM analysis. Figure 2a shows that s-TiO_2_ nanoparticles have good dispersion with size of about 10–20 nm. As shown in Figure 2b, s-TiO_2_ nanoparticles can be observed to be assembled by multiple nanocrystals with 3–5 nm. Figure 2c shows that a clear anatase–rutile interface exists between two nanocrystals, in which the anatase phase plane (101) has a lattice spacing of ~0.352 nm, while the lattice spacing of rutile phase plane (110) is ~0.320 nm [39]. In Figure 2d–f, the t-TiO_2_ sample has a smaller nanocrystal diameter and a narrower size distribution, and the growth directions are different, but all nanograins are anatase phases. For t-TiO_2_ exists a significant agglomeration, which results in the lower BET surface value in comparison with s-TiO_2_. This may be ascribed to the influence of alcohol reagent and crystal nucleation rate during the reflux reaction. The molecular chain of sec-butanol is so long that the hydroxyl groups are separated from each other, and its reactivity is not as good as the hydroxyl groups in tert-butanol. Moreover, the surface energy of the anatase phase of TiO_2_ nanocrystals is lower than that of the rutile phase, which implies that the more stable anatase phase in two systems will preferentially form. Therefore, in the sec-butanol reagent, the initial anatase-phase in s-TiO_2_ nanocrystal can grow more dispersed with a larger growth space and a slower growth rate. Then, the rutile-phase of s-TiO_2_ nucleates on the surface or interface of the initial anatase-phase nanocrystal, and finally forms a mixed-phase crystal [40,41,42,43]. On the contrary, in the tert-butanol reagent, the anatase grains may agglomerate seriously and the gaps between the grains are insufficient, which result in no rutile phase in the t-TiO_2_ nanocrystals. As a comparison, the P25 sample exhibits an average particle size of ~30–50 nm in Figure 2g–i.

**Figure 3:**
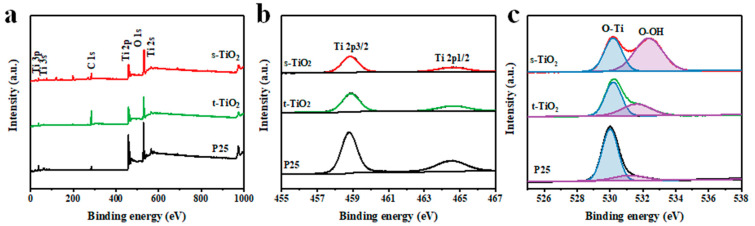
(**a**) XPS overall, (**b**) Ti 2p, and (**c**) O 1s spectrum of s-TiO_2_, t-TiO_2_, and P25.

XPS measurement can analyze elemental composition and chemical state. The XPS spectrum in Figure 3a shows that the three samples contain the elements of titanium and oxygen from TiO_2_ nanocrystals. In Figure 3b, the two peaks near 459 and 465 eV for s-TiO_2_ correspond to Ti 2p_3/2_ and Ti 2p_1/2_ respectively, which both belong to the Ti (IV) ion of TiO_2_ [36]. The Ti 2p spectrum has no obvious difference with the other two samples of t-TiO_2_ and P25. In the O 1s XPS spectrum for s-TiO_2_ (Figure 3c), the peak near 530 eV is derived from the lattice oxygen, and the peak near 533 eV is ascribed to the surface-bridged hydroxyl [11]. The intensity of the bridging hydroxyl peak of s-TiO_2_ far exceeds the intensity of t-TiO_2_ and P25, which means that the photogenerated holes and these hydroxyl groups have a greatly enhanced ability to generate strong oxidizing free radicals (OH) on the surface of s-TiO_2_ [44]. These strong oxidizing free radicals can directly oxidize organic matter into small inorganic molecules, such as CO_2_ and H_2_O, thereby facilitating the occurrence of photocatalytic degradation reactions.

### 3.2. Photocatalytic Performance

**Figure 4:**
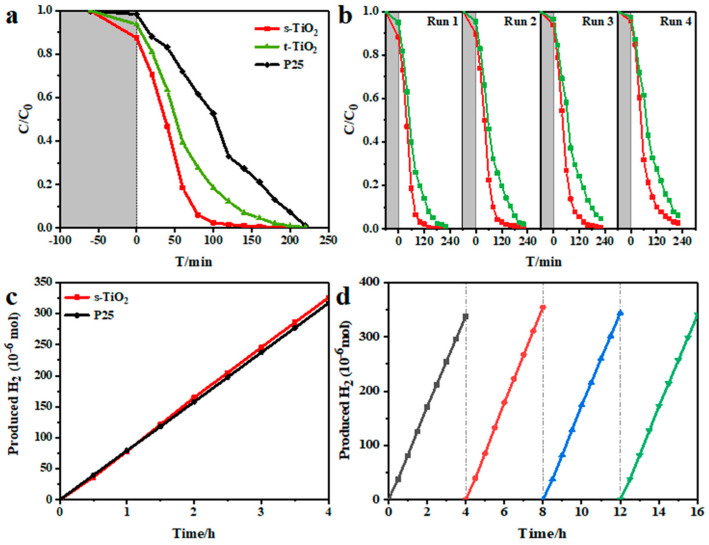
(**a**) Ultraviolet photocatalytic degradation performance, (**b**) photocatalytic degradation cycle stability test, (**c**) photocatalytic H_2_ evolution rate, and (**d**) photocatalytic cycle hydrogen production performance of s-TiO_2_.

The ultraviolet photocatalytic degradation performance of three TiO_2_ samples is shown in Figure 4a. Compared with t-TiO_2_ and P25, s-TiO_2_ exhibits excellent degradation ability. After 40 min, the concentration drops to half of the initial value, and the residual is only 1% of the initial concentration during another 60 min.

The first-order rate constant, k, of the reaction can be obtained from the following first-order non-linear dynamic equation:

Ln(C_0_/C) = −kt (3)

In Equation (3), C_0_ represents the original dye concentration before degradation, and C represents the dye concentration at the reaction time t (min). The first-order rate constant k of s-TiO_2_, t-TiO_2_, and P25 are 0.0352, 0.0164, and 0.0094 min^−1^, respectively. It can be seen that the photocatalytic degradation performance of s-TiO_2_ and t-TiO_2_ is 3.74 times and 1.76 times that of P25. The cycling stability is shown in Figure 4b, which exhibits that the photocatalytic degradation of the s-TiO_2_ and t-TiO_2_ nanocrystals can still be maintained very well after four cycles. However, as shown in Figure 4c, s-TiO_2_ has no obvious advantage over P25 in the performance of photocatalytic decomposition of water to produce hydrogen. As shown in Figure 4d, s-TiO_2_ has a stable performance of photocatalytic decomposition of water to produce hydrogen. After three cycles of testing, its hydrogen production performance remains basically unchanged.

### 3.3. Material Photocatalytic Mechanism Characterization Test

**Figure 5:**
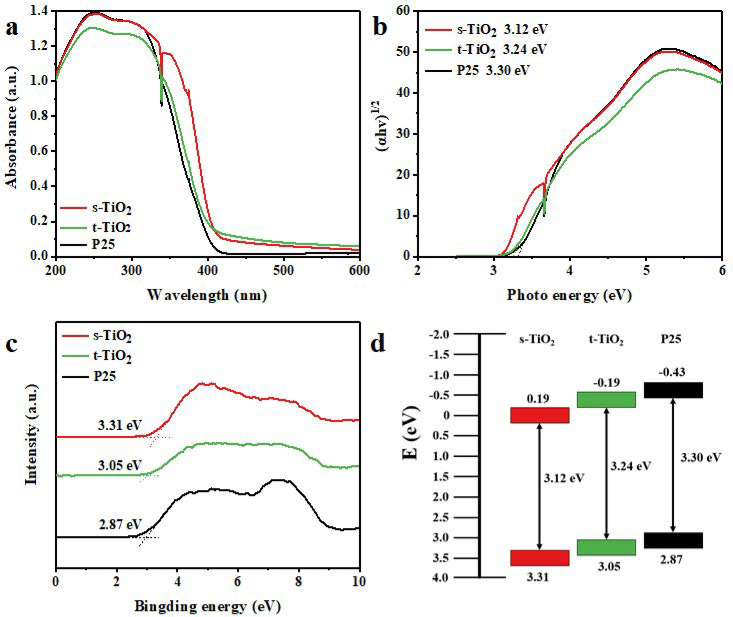
(**a**) UV-Vis DRS spectra, (**b**) Tauc bandgap diagram, (**c**) VB XPS spectra, and (**d**) band structure alignments of s-TiO_2_, t-TiO_2_, and P25.

The UV-Vis diffuse reflectance spectra (DRS) can be used to determine the light absorption performance and bandgap of catalysts. As shown in Figure 5a, compared with P25, s-TiO_2_ shows the wider background absorption at wavelengths greater than 330 nm, while t-TiO_2_ has weaker absorption in the ultraviolet region. Therefore, s-TiO_2_ has the largest light absorption range among the three samples. The Tauc plots of (αhv) 1/2 and hv of TiO_2_ nanocrystals shown in Figure 5b are obtained from the transformation of the curves in Figure 5a, showing that the indirect bandgap energies of s-TiO_2_ and t-TiO_2_ are 3.12 and 3.24 eV, respectively. Although s-TiO_2_ is composed of two crystal phases including anatase and rutile, its indirect bandgap energy is almost the same as that of pure rutile phase TiO_2_ (3.11 eV) [45]. Meanwhile, t-TiO_2_ is only composed of anatase phase and its bandgap energy is consistent with that of the pure anatase phase TiO_2_ (3.26 eV). The indirect bandgap energy of the s-TiO_2_ nanocrystals is far lower than that of t-TiO_2_ and P25 (3.30 eV), which means that the incident electron energy that excites the photocatalyst to generate photogenerated electron–hole pairs is reduced; that is to say, the light absorption range expansion may be one of the reasons for the improved photocatalytic degradation performance. Characterizations of the electronic structure can be performed by valence band (VB) XPS. The VB XPS spectrum (Figure 5c) shows that the maximum VB of the s-TiO_2_, t-TiO_2_, and P25 are 3.31, 3.05, and 2.87 eV respectively, which means that the valence band potential of s-TiO_2_ is more positive than those of the other two samples. The electrons in the valence band are excited to the conduction band and become photogenerated electrons after the semiconductor absorbs light, while leaving the same amount of photogenerated holes in the valence band. The valence band potential is based on the standard hydrogen electrode potential. The valence band potential of s-TiO_2_ is larger, which means that the holes in the valence band have stronger oxidation ability for facilitating the degradation process [36]. Combining UV-Vis absorption and VB XPS spectrum results in the bandgap diagram shown in Figure 5d, compared with t-TiO_2_ (−0.19 eV) and P25 (−0.43 eV), the conduction band potentials of s-TiO_2_ is 0.19 eV. A more positive conduction band potential means that the reduction ability of photogenerated electrons on the conduction band is weakened, but the degradation of RhB dye in this study depends on the oxidation performance of the photocatalyst. Therefore, due to the expansion of the light absorption range and the increased valence band potential, the photocatalytic degradation performance of s-TiO_2_ could be greatly improved compared with t-TiO_2_ and P25.

**Figure 6:**
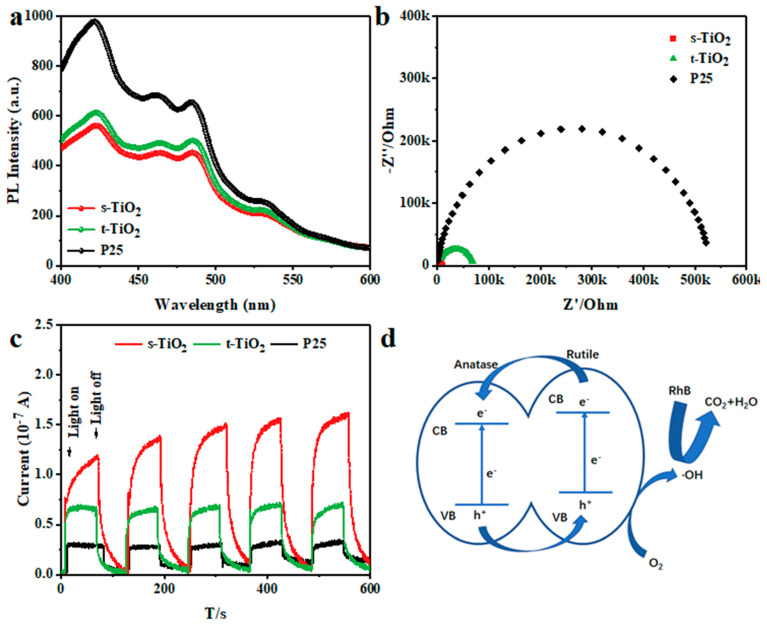
(**a**) PL spectrum, (**b**) EIS Nyquist plot, (**c**) photoelectrochemical responses, and (**d**) band structure alignments of s-TiO_2_ and P25.

The photoluminescence spectrum (PL) reveals the recombination efficiency of free carriers. In Figure 6a, strong peaks near 425 nm derive from the direct bandgap transition, and the small PL peaks between 440 and 500 nm stem from the excitonic PL caused by defects and oxygen vacancies in the samples [24]. After light absorption, the electrons and holes generated by charge separation migrate inside the photocatalyst, and then move to the surface, causing the redox reaction at the solid–liquid interface between photocatalyst surface and solution. The nanocrystal diameters of s-TiO_2_ and t-TiO_2_ are significantly smaller than that of P25, which means that the path of electrons and holes moving to the surface is much shorter. The number of electron–hole recombination occurring inside the semiconductor is reduced, and more carriers can diffuse to the surface of the semiconductor to promote degradation. The significantly increased ratio of rutile in s-TiO_2_ mixed nanocrystal is beneficial to the increase in the number of energy-level interlaced interfaces. The carriers tend to transfer between two phases, thereby promoting carrier separation and reducing the possibility of internal electron–hole recombination [36]. The PL intensity of s-TiO_2_ is even lower than that of t-TiO_2_ and P25, which undoubtedly proves the advantage of the mixed crystal structure with nearly equal rutile/anatase ratio.

In addition, the electrochemical impendence spectra (EIS) are shown in Figure 6b. The three kinds of TiO_2_ all show a typical semicircle curve, and the arc diameter of s-TiO_2_ is the smallest. The diameter of the semicircle corresponds to the charge transfer resistance, which can reflect the recombination and transfer behavior of carriers [46]. The charge transfer resistance of s-TiO_2_ is ~9 kΩ, which is much smaller than t-TiO_2_ (~71 kΩ) and P25 (~533 kΩ). The large specific surface area leads to a significant increase in surface bridged hydroxyl groups in s-TiO_2_, which may promote charge transfer [8]. The charge transfer resistance can strongly influence the photocatalytic performance. In fact, the smaller charge transfer resistance corresponds to the higher degradation activity. That is, the s-TiO_2_ is the best photocatalyst for degradation, followed by t-TiO_2_ and P25.

The intensity of the photoelectric response current (Figure 6c) can reflect the generation and transmission rate of photogenerated carriers [19]. The three kinds of TiO_2_ have relatively stable instantaneous photocurrent response under continuous and non-illuminated conditions. The small nanocrystal size can shorten the distance of carriers and promote diffusion from the inside to the surface of the catalyst, so s-TiO_2_ shows the strongest photocurrent intensity among them, which is close to 1.4 × 10^−7^ A. In addition, the mixed nanocrystal structure can not only promote the transfer of photogenerated carriers between two phases, thereby prolonging the life of the carriers, but also increase the number of defects that are beneficial to photocatalysis to a certain extent [12,47,48]. It is worth noting that P25 is also a mixed crystal structure, and its photoelectric response signal is not as good as s-TiO_2_. Similar with photocatalytic activity, it suggests that the synergistic effect of small particle size and mixed crystal structure contributes to the carrier transport rate.

Finally, we summarize the photocatalytic mechanism of s-TiO_2_. On one hand, the small particle size is conducive to the movement of the photogenerated electron–hole pairs from the generation site to the solid surface, reducing the recombination of internal carriers [33,49]. The large specific surface area of s-TiO_2_ can contribute to the increase of hydroxyl groups on the surface and thus the improvement of photocatalytic degradation. On the other hand, as shown in Figure 6d, the presence of mixed crystal phases with similar content in s-TiO_2_ helps to form more energy-level interleaved interfaces, and photogenerated electrons tend to transfer from the rutile phase to the anatase phase, which promotes charge separation [50,51]. Therefore, the possibility of photogenerated electron–hole pair recombination is reduced, the life of photogenerated carriers is prolonged, and the photocatalytic reaction activity is improved [14,24,33,49].

## 4. Conclusions

In summary, we have successfully synthesized novel TiO_2_ nanocrystals with rutile-rich crystal phase structure through the one-step condensing reflux method. The improvement of photocatalytic degradation for TiO_2_ mixed nanocrystals can be mainly attributed to the numerous energy-level staggered interfaces between two phases, large specific surface area, and significantly increased surface bridged hydroxyl groups. This research may help to further study the effect of multi-phase nanocrystalline semiconductor on the photocatalysis.

## Data Availability

No new data were created or analyzed in this study. Data sharing is not applicable to this article.
